# Systemic desensitization through TRPA1 channels by capsazepine and mustard oil - a novel strategy against inflammation and pain

**DOI:** 10.1038/srep28621

**Published:** 2016-06-30

**Authors:** Katrin Kistner, Norbert Siklosi, Alexandru Babes, Mohammad Khalil, Tudor Selescu, Katharina Zimmermann, Stefan Wirtz, Christoph Becker, Markus F. Neurath, Peter W. Reeh, Matthias A. Engel

**Affiliations:** 1 Institute of Physiology and Pathophysiology, Friedrich-Alexander-Universität Erlangen-Nürnberg, Universitätsstr. 17, 91054 Erlangen, Germany; 2 University of Bucharest Department of Physiology, Faculty of Biology, Splaiul Independentei 91-95, 050095 Bucharest, Romania; 3 Department of Medicine 1, Universitätsklinikum Erlangen, Ulmenweg 18, 91054 Erlangen, Germany; 4 Department of Anaesthesiology, Universitätsklinikum Erlangen, Krankenhausstr. 12, 91054 Erlangen, Germany

## Abstract

We demonstrate a novel dual strategy against inflammation and pain through body-wide desensitization of nociceptors via TRPA1. Attenuation of experimental colitis by capsazepine (CPZ) has long been attributed to its antagonistic action on TRPV1 and associated inhibition of neurogenic inflammation. In contrast, we found that CPZ exerts its anti-inflammatory effects via profound desensitization of TRPA1. Micromolar CPZ induced calcium influx in isolated dorsal root ganglion (DRG) neurons from wild-type (WT) but not TRPA1-deficient mice. CPZ-induced calcium transients in human TRPA1-expressing HEK293t cells were blocked by the selective TRPA1 antagonists HC 030031 and A967079 and involved three cysteine residues in the N-terminal domain. Intriguingly, both colonic enemas and drinking water with CPZ led to profound systemic hypoalgesia in WT and TRPV1^−/−^ but not TRPA1^−/−^ mice. These findings may guide the development of a novel class of disease-modifying drugs with anti-inflammatory and anti-nociceptive effects.

Sensory neurons of nociceptive character, which encode chemical, thermal, and mechanical stimuli and release neuropeptides, pervade the whole mammalian body. These nerve fibers are equipped with polymodal ion channel receptors, which are members of a large family, including the two major irritant receptor-channels transient receptor potential ankyrin 1 (TRPA1) and transient receptor potential vanilloid 1 (TRPV1)^1^,^2^,^3^,^4^. Prototypical activators of these receptors are the pungent ingredients of mustard oil (allyl isothiocyanate, AITC) and chili peppers (capsaicin, CAP), respectively (1). Both channels are involved in transduction of painful conditions and sensitization of each receptor leads to hyperalgesia to various stimuli and allodynia^2^,^3^,^4^. Their pathophysiological role is based on their capacity to be activated by various mediators and metabolites produced under inflammatory conditions. TRPV1 is activated by protons (tissue acidosis) and is indirectly sensitized by bradykinin and prostaglandins^5^. Protons and various lipid peroxidation products such as 4-hydroxynonenal that are produced in states of inflammation and oxidative stress activate the human TRPA1 receptor channel^1^. Both ion channels have also been shown to be involved in the pathogenesis of various inflammatory diseases including human inflammatory bowel diseases and experimental colitis^6^,^7^,^8^,^9^,^10^,^11^. Antagonism or genetic deletion of either channel also reduced inflammation in models of asthma and dermatitis, supposedly through inhibition of neuropeptide release^12^,^13^,^14^,^15^. This anti-inflammatory mechanism has also been attributed to capsazepine (CPZ), the prototypical TRPV1 antagonist, which when repeatedly administered via enema, attenuated experimental colitis^7^,^8^. However, we showed that TRPA1 plays a far more important role than TRPV1 in colitis^6^. To resolve this discrepancy, we sought to determine whether TRPA1 is also involved in colitis protection through unknown, off-target effects of CPZ.

In this study we show that TRPA1 agonism, rather than TRPV1 inhibition, in colonic sensory neurons is the key initial step in colitis protection by CPZ enemas. Intriguingly, colonic and systemic administration of CPZ or AITC induced a profound body-wide, TRPA1-mediated desensitization of nociception in mice. Chronic administration of these TRPA1 agonists was well tolerated. Thus, systemic desensitization through TRPA1 may provide a novel strategy for medicinal treatment of various chronic inflammatory and pain states.

## Results

### CPZ attenuates colitis independently of TRPV1

**Figure 1:**
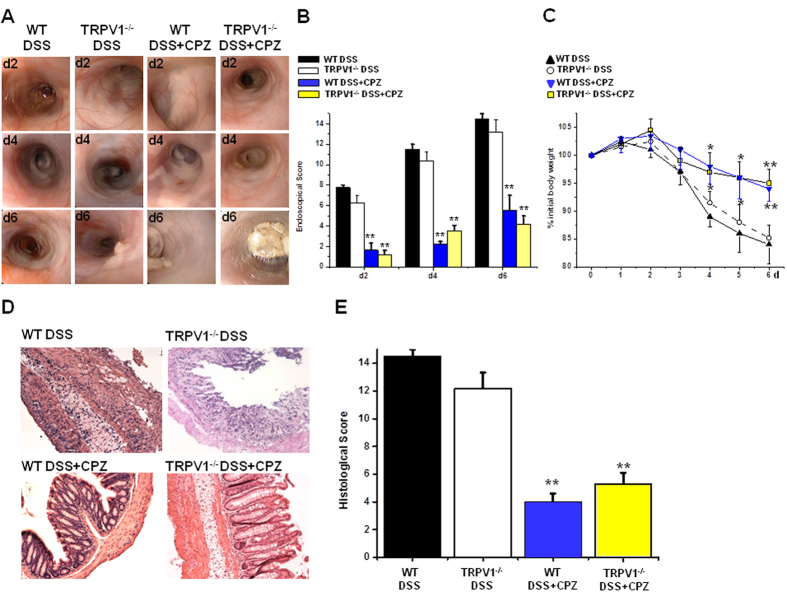
Capsazepine (CPZ) enemas attenuate murine DSS colitis independently of TRPV1. (**A**) Representative colonoscopy photographs during the course of DSS (5%) colitis at days 2, 4 and 6 (d2, d4, d6) (each group n = 8). Comparison of WT and TRPV1-deficient mice without CPZ (531 μM) enemas with WT and TRPV1^−/−^ mice that were treated twice daily with CPZ enemas from day 0. (**B**) Endoscopic score (Mann Whitney U-test) (**C**) course of body weight analysis of variance (ANOVA) followed by Fisher’s LSD test, (**D**) histological examination (HE staining) and (**E**) histological score (Mann Whitney U-test) in the different groups. All *P < 0.05, **P < 0.01.

To challenge the mechanism by which CPZ enemas attenuate experimental colitis, we induced dextran sulphate sodium (DSS) colitis in wild-type (WT) and TRPV1-deficient mice (each group n = 8). Although conflicting reports exist, TRPV1-deficient mice developed DSS colitis and weight loss to the same degree as the congenic WT mice in our laboratory, which complies with our results from the model of TNBS colitis that we had previously published^7^,^8^,^9^,^10^,^11^,^12^. For CPZ enema treatments, we used the same concentration of CPZ (531 μM) that was previously reported to attenuate DSS (5%) colitis in rats^8^. The course of colitis was monitored daily by body weight measurements and endoscopy. Twice-daily applications of CPZ (531 μM) enemas attenuated DSS colitis to the same degree in both WT and TRPV1^−/−^ mice, which was reflected by an improved endoscopic score and reduced loss of body weight (Fig. 1A–C). H&E stains from the distal colon at the end of the 7 day DSS experiment revealed destroyed mucosal tissue architecture with numerous infiltrating immune cells in the colons of controls. This was in stark contrast to a widely intact mucosa and the absence of significant immune cell infiltration in the colons of CPZ-treated mice of both genotypes (Fig. 1D). In accordance with these findings, the histological score was strongly reduced in the CPZ-treated mice of both genotypes (Fig. 1E).

### CPZ activates TRPA1

The *in vivo* observation that CPZ enemas effectively inhibited colitis in TRPV1 null mutant mice imposed the question on TRPV1-independent neuronal effects of CPZ. Since TRPA1 had been shown to be crucial in various models of inflammation including colitis^6^,^12^,^14^, we tested whether CPZ acts on TRPA1.

#### CPZ-induced ionic currents through hTRPA1

**Figure 2:**
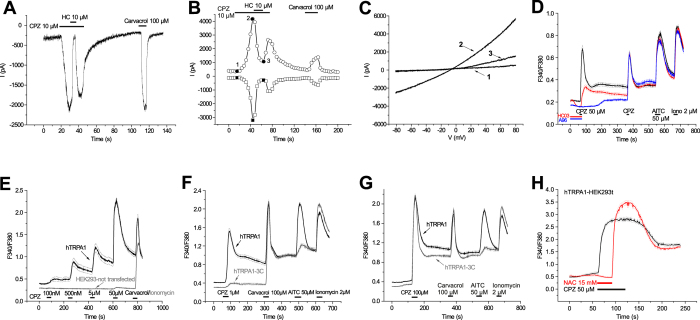
Capsazepine (CPZ) activates heterologously expressed human TRPA1. (**A**) CPZ (10 μM) evokes hTRPA1-mediated currents in transfected HEK293t cells. Note the complete inhibition of currents by the selective TRPA1 antagonist HC030031 (HC, 10 μM). (**B**) Representative ramp currents evoked in a hTRPA1-transfected HEK293 cell by 400 ms voltage ramps from −100 to +100 mV applied every 4 s. Each symbol represents the current amplitude at −80 mV (squares) and +80 mV (circles). The CPZ-induced current was strongly inhibited by HC. (**C**) Examples of individual ramp currents corresponding to the filled symbols and numbers in B. (**D**) CPZ (50 μM, 10 s) evokes large calcium transients in hTRPA1-HEK293 cells (black trace, n = 128). HC (20 μM; red trace, n = 209) and A-967079 (10 μM; blue trace, n = 140) completely inhibited the response to CPZ. Data represent means (straight lines) ± SEMs (dotted lines). (**E**) The activation of hTRPA1 by CPZ (10 μM) is concentration-dependent. Black trace: hTRPA1-HEK293 cells (n = 52) were stimulated by increasing concentrations of CPZ for 20 s at 3 min-intervals. Gray trace: untransfected HEK293 cells (n = 101) were subjected to the same CPZ concentrations. (**F**) Activation of hTRPA1 by CPZ (1 μM) involves three critical cysteines in the N-terminus of the channel. Black trace: response of n = 123 HEK293 cells expressing WT hTRPA1 to successive applications of CPZ (1 μM), carvacrol (100 μM) and allyl isothiocyanate (AITC, 50 μM). Gray trace: response of n = 165 HEK293 cells expressing the mutant hTRPA1-3C to the same sequence of stimuli. Note the substantial reduction in the response of the hTRPA1-3C mutant to CPZ and AITC, but not to carvacrol. (**G**) The difference in CPZ sensitivity between genotypes was abolished at 100 μM CPZ. (**H**) Activation of hTRPA1 by CPZ can be prevented by the scavenger N-acetyl cysteine (NAC). Black trace: in control experiments hTRPA1-HEK293 cells were challenged with CPZ (50 μM; 60 s; n = 74). Red trace: in a separate experiment cells were first exposed for 30 s to a combination of CPZ (50 μM) and NAC (15 mM), immediately followed by CPZ alone for another 30 s (n = 83).

Patch clamp experiments were carried out in voltage clamp mode on HEK293t cells expressing recombinant hTRPA1 (hTRPA1-HEK293t cells). At a holding potential of −60 mV, CPZ (10 μM) induced an inward current that was almost completely inhibited (93% inhibition, n = 5) by the selective TRPA1 antagonist HC-030031 (HC, 10 μM) (Fig. 2A). In all cells in which a CPZ-induced inward current was recorded, carvacrol (100 μM) an established TRPA1 agonist, also evoked large inward currents at the same holding potential, demonstrating functional expression of hTRPA1. These hTRPA1-HEK293 cells were also subjected to voltage ramps from −100 to +100 mV of 400 ms duration every 4 s (Fig. 2B). The CPZ-induced, current-voltage relationship displayed slightly outward rectification and a reversal potential close to 0 mV (Fig. 2C). The currents evoked by CPZ (10 μM) were inhibited by HC (10 μM). The effect was more pronounced at negative potentials (89% ± 5% inhibition at −80 mV, mean ± SD, n = 7) than at positive potentials (80% ± 9% inhibition at +80 mV).

#### CPZ-induced calcium-influx through hTRPA1

Selective action of CPZ on TRPA1 was confirmed by employing the calcium microfluorimetry technique. hTRPA1-HEK293 cells were stimulated by two applications of CPZ (50 μM) for 10 s at 5-min intervals (Fig. 2D). The selective antagonists HC (20 μM) and A-967079 (10 μM) were applied for 1 min before and during the first CPZ challenge. The CPZ response was completely abolished by both antagonists. It is worth noting that the removal of HC led to a calcium influx, probably due to residual CPZ action, while this off effect was absent in the case of A-967079 which may detach more slowly (Fig. 2D). We then analyzed concentration-dependency of CPZ effects. Increasing CPZ concentrations (100 nM, 500 nM, 5 μM, and 50 μM) were applied to hTRPA1-HEK293 cells for 20 s each at 3-min intervals. Beginning with 100 nM, all concentrations of CPZ evoked calcium transients with increasingly larger amplitudes (Fig. 2E). Carvacrol (100 μM) was applied at the end of the experiment to control for functional TRPA1 expression, but the carvacrol response after 50 μM CPZ was conspicuously small, suggesting cross-desensitization. Untransfected HEK293 cells were subjected to the same CPZ applications and only the highest concentration tested (50 μM) induced a minimal increase in calcium. We expected that CPZ would engage the three critical cysteine residues in the N-terminal domain of the channel because it is an electrophilic compound. HEK293 cells expressing WT hTRPA1 and cells expressing the triple cysteine mutant hTRPA1-3C (C621S, C641S, C665S) were subjected to CPZ application (1 μM, 20 s) followed by the non-electrophilic agonist carvacrol (100 μM, 20 s) and the highly electrophilic AITC (50 μM, 30 s). Ionomycin was applied at the end of the experiment as a positive control. The amplitude of the calcium transient evoked by 1 μM CPZ was substantially reduced (>80%) in cells expressing hTRPA1-3C compared with cells expressing WT hTRPA1 (Fig. 2F), while the difference in CPZ sensitivity between the genotypes was abolished at 100 μM CPZ concentration (Fig. 2G). The AITC response was decreased in the mutant cells, whereas carvacrol evoked large calcium ion transients in both WT and 3C mutants. Taken together, these cell responses indicate that the relatively high potency of CPZ depends on the three critical cysteines. However, when the concentration of CPZ is 100-times higher other binding sites take over the activation of hTRPA1. This high concentration of the lipophilic CPZ could also interact with the cellular lipid membrane, indirectly activating TRPA1, as previously shown for the lipid A component of lipopolysaccharides^16^. Moreover, in the presence of the electrophile scavenger N-acetyl cysteine (NAC) at a saturating concentration (15 mM) 50 μM CPZ was unable to elicit any response. Upon removal of NAC, CPZ evoked large calcium transients in the hTRPA1-transfected HEK293t cells (Fig. 2H) which confirms that CPZ acts as an electrophilic agonist for hTRPA1.

#### CPZ activates a subpopulation of AITC-sensitive dorsal root ganglion (DRG) neurons

**Figure 3:**
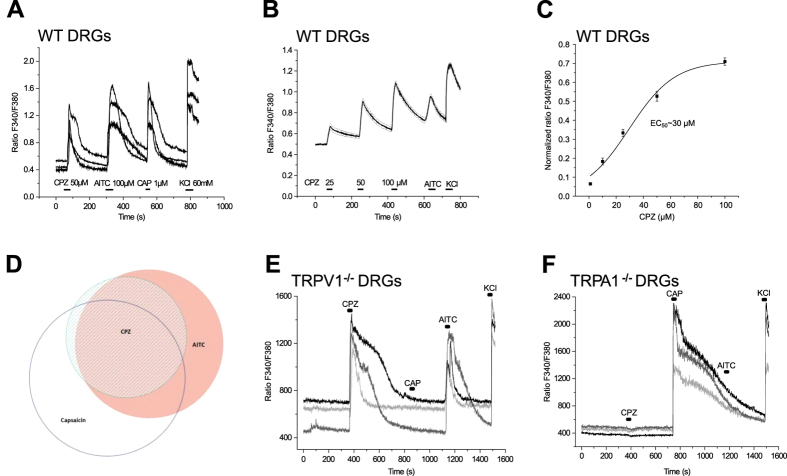
Capsazepine (CPZ) activates murine TRPA1 in dorsal root ganglion neurons. (**A**) CPZ (50 μM) activates a subpopulation of AITC (100 μM)-sensitive mouse dorsal root ganglion (DRG) neurons. Individual responses from three different AITC- and capsaicin (CAP, 1 μM)-sensitive DRG neurons that were activated by CPZ. CPZ was applied for 20 s, AITC for 30 s and CAP for 10 s at intervals of 4 min allowing recovery. KCl (60 mM) was applied at the end of the experiment to to ensure viability of cultured neurons. (**B**) Averaged response of n = 135 AITC-sensitive neurons to three different concentrations of CPZ (25, 50, 100 μM, 20 s each). Note the concentration-dependence of the amplitude of Ca^2+^ transients. Straight traces represent mean and dotted traces represent SEMs. (**C**) Concentration-dependent increase of CPZ-induced [Ca^2+^]_i_ in AITC-sensitive DRG neurons normalized to a depolarizing stimulus with KCl (60 mM). The EC_50_ of ∼30 μM was calculated by fitting to the Dose-Response function. Data are representative of two sets of experiments and show means ± SEM; for CPZ 1, 10, 25 μM: n = 169, for CPZ 25, 50, 100 μM: n = 138. (**D**) Illustrating populations of CPZ-, AITC-, and CAP-sensitive DRG neurons and their overlap. In a total of 906 imaged neurons (selected by their response to KCl), 200 (22%) were activated by CPZ (50 μM), 301 (33%) by AITC (100 μM) and 323 (36%) by CAP (1 μM) (detailed analysis of fractions, see *SI* Figure Legend 3). (**E**) CPZ (100 μM, 20 s) activates AITC (100 μM, 20 s)-sensitive DRG neurons from TRPV1-deficient mice. Individual responses from different AITC-sensitive neurons that were activated by CPZ. About 90% (509/572 cells) of the TRPV1-deficient DRG neurons that were AITC-sensitive were responsive to CPZ. CAP (1 μM, 10 s) did not induce Ca^2+^ transients in TRPV1^−/−^ neurons. (**F**) Lack of CPZ (100 μM)-induced Ca^2+^ influx in TRPA1-deficient DRG neurons. Individual responses from different CAP (1 μM)-sensitive DRG neurons (out of 448 neurons tested) that were neither activated by CPZ nor AITC (10 μM).

DRG neurons were maintained in primary culture and investigated by calcium microfluorimetry. Cells were stimulated by CPZ (50 μM, 20 s), followed by AITC (100 μM, 30 s), capsaicin (CAP, 1 μM, 10 s) and KCl (60 mM, 30 s). Figure 3A illustrates examples of DRG neurons responding to all these four stimuli. A typical fraction of DRG neurons was activated by AITC (301 of 906 neurons, 33%, n = 8 mice) indicating functional expression of TRPA1. A subpopulation of these AITC-sensitive neurons was also activated by CPZ (185 of 301 neurons, 61%). The sensitivity to CPZ was almost completely restricted to AITC-responsive neurons. Of altogether 200 CPZ-sensitive neurons, 185 (93%) were also activated by AITC, indicating a strong concordance of sensitivities to CPZ and AITC (Fig. 3). As in the case of hTRPA1-expressing HEK293 cells, the calcium transients evoked by CPZ in DRG neurons were concentration-dependent with an EC_50_ value estimated to 30 μM CPZ and the small AITC response after 100 μM CPZ again suggested cross-desensitization (Fig. 3B,C). Figure 3D shows the overlap of CPZ, CAP, and AITC-responsiveness in WT DRG neurons at given concentrations: 14% of the DRG neurons responded to AITC but not to CAP (125/906), 16% responded to CAP but not to AITC, whereas CPZ induced calcium transients in 78% of the neurons that responded to both AITC and CAP (137 of 176). To demonstrate the specificity of the observed effects, TRPV1^−/−^ and TRPA1^−/−^ DRG neurons were exposed to the above protocol. About 90% of the TRPV1-deficient DRG neurons that were AITC-sensitive also responded to CPZ (509/572 cells), while none of the 448 TRPA1-deficient DRG neurons tested showed calcium influx in response to CPZ (100 μM) as illustrated by representative examples in Fig. 3E,F.

### Systemic desensitization through TRPA1 attenuates pain

**Figure 4:**
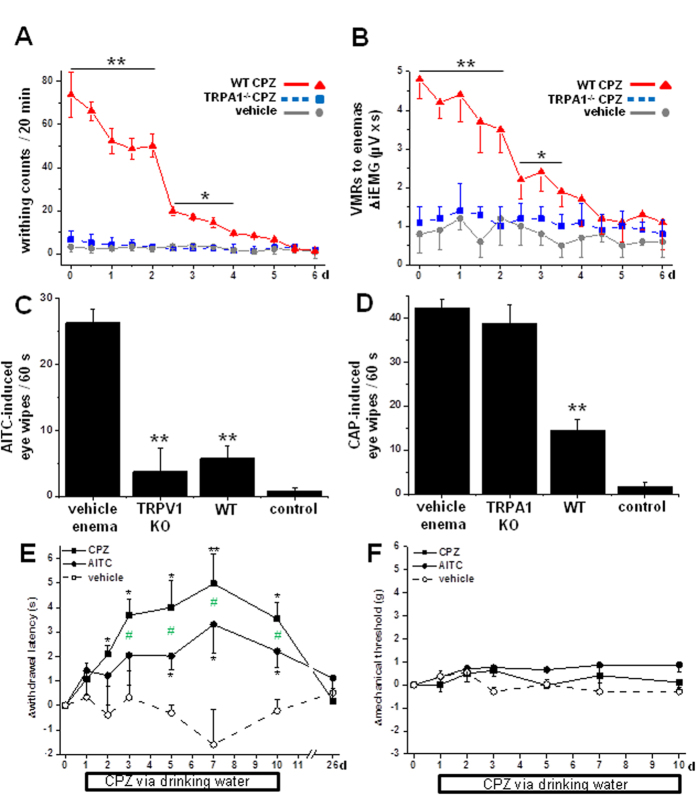
Capsazepine enemas desensitize local and distant pain responses. (**A**) Acute writhing reactions in response to CPZ (531 μM) enemas during the first 5 min after application. Repeated CPZ enemas (twice daily) led to a progressive decrease of writhing responses. A dramatic step reduction was observed after 5 enemas in WT mice. By contrast, TRPA1^−/−^ mice treated with CPZ (531 μM) or WT mice treated with vehicle (PBS) enemas displayed only few writhing that occurred within the first 30 s (each n = 6). (**B**) Visceromotor responses (VMRs) to CPZ enemas. CPZ enemas twice daily led to a continuous decrease of VMRs in WT mice, similar to the course of writhing reactions. VMRs in response to vehicle were not significantly different from those to CPZ enemas in TRPA1^−/−^ (both n = 6). (**A**,**B**) WT CPZ group compared to vehicle group. (**C**) Repeated CPZ enemas attenuate eye-wipe behavior to AITC (100 μM). Both WT and TRPV1^−/−^ mice treated with CPZ enemas showed a profound reduction of eye-wipe counts compared with vehicle (both n = 6). Controls were WT mice without enemas receiving vehicle (PBS) drops into the eye. (**D**) CPZ enemas attenuate eye-wipe behavior to CAP (1 mM). WT mice treated with vehicle and TRPA1^−/−^ mice treated with CPZ enemas twice daily for 7 d showed numerous eye-wipe reactions to CAP instillation into the eye, tested 12 h after the last CPZ enema (both n = 6). WT mice treated with CPZ enemas showed profound reduction of eye wipe counts. (**E**) Thermal and (**F**) mechanical withdrawal thresholds of both hindpaws during the course of oral CPZ (531 μM) or AITC (500 μM) medication via drinking water. (**E**) CPZ and AITC over 10 d compared to the vehicle-treated group induced a progressive increase during 3–5 d in withdrawal latencies to radiant heat stimulation, which normalized within 2 weeks of wash-out (each n = 6). (**F**) Mechanical thresholds to stimulation with an electrodynamic von Frey filament showed no systematic trend under both compounds (each n = 6). All repeated measures ANOVA and Dunnett’s post-test, except in (**C**,**D**) Mann Whitney U-test.

After we had discovered that CPZ is a potent TRPA1 agonist, we understood why the first CPZ enemas (twice daily) were obviously painful in the WT mice. We then quantified the CPZ (531 μM) enema-induced nocifensive behavior in healthy animals (each n = 6) by counting the writhing reactions and recording visceromotor reflex responses (VMRs) through the integrated electromyography (EMG) of the abdominal muscle wall (Fig. 4A,B). During the course of repeating the twice-daily CPZ treatments, we noted that the TRPA1 knockouts did not show pain-related behavior at any time. In addition, the WT mice presented a progressive decrease of initially strong pain responses with a steep decline around day 3 which led to a finally complete desensitization in both nocifensive parameters. This loss of colonic pain perception in otherwise normal mice raised the expectation that the enemas might have delivered CPZ a pharmacodynamic amount sufficient to induce systemic hypoalgesia. To test this hypothesis, we employed the eye-wipe test using AITC (100 μM) and CAP (1 mM) (Fig. 4C,D) (n = 6). Both tests showed a distinct attenuation of eye-wipe counts in the WT mice that had been treated with CPZ enemas (until 12–16 h before the test). These effects were most probably mediated by TRPA1 desensitization rather than TRPV1 block, since TRPV1-deficient mice were insensitive to mustard oil (AITC, 100 μM) instillation into the eye to the same degree as WT mice (Fig. 4C). Vice versa, CPZ enemas were ineffective in TRPA1^−/−^ mice, when these mice were challenged by CAP instillations into the eye (Fig. 4D).

Since repeated enemas are an unpleasant route of administration, we tested for a possible anti-nociceptive effect of peroral CPZ (531 μM) in the drinking water. The drinking regimen over 10 days was well tolerated with no obvious adverse effects occurring. During the course of continuous oral CPZ administration paw-withdrawal latency to radiant heat stimulation (Hargreaves´ method) progressively increased in both hindpaws (Fig. 4E). The tolerance time was about doubled at day 7 and remained significantly elevated from day 2 to 10. After 2 weeks of recovery with pure drinking water, withdrawal latencies had returned to baseline level. The mechanical responsiveness to stimulation with the linearly increasing force of an electrodynamic von Frey filament was not significantly affected during the ten days of CPZ drinking (Fig. 4F). The question arose whether the heat and chemical hypoalgesia represented a class effect of desensitizing TRPA1 agonists or whether it was specific for CPZ. Thus we administered AITC in a similar and well tolerated concentration (500 μM) via the drinking water. The same dose regimen for AITC as described for CPZ resulted in a progressive increase of paw-withdrawal latency to radiant heat stimulation that was significantly smaller than the one induced by CPZ (Fig. 4E). Mechanical responsiveness was not changed under the AITC drinking regimen (Fig. 4F).

### CPZ causes sustained desensitization of TRPA1/TRPV1 expressing peptidergic sensory neurons

**Figure 5:**
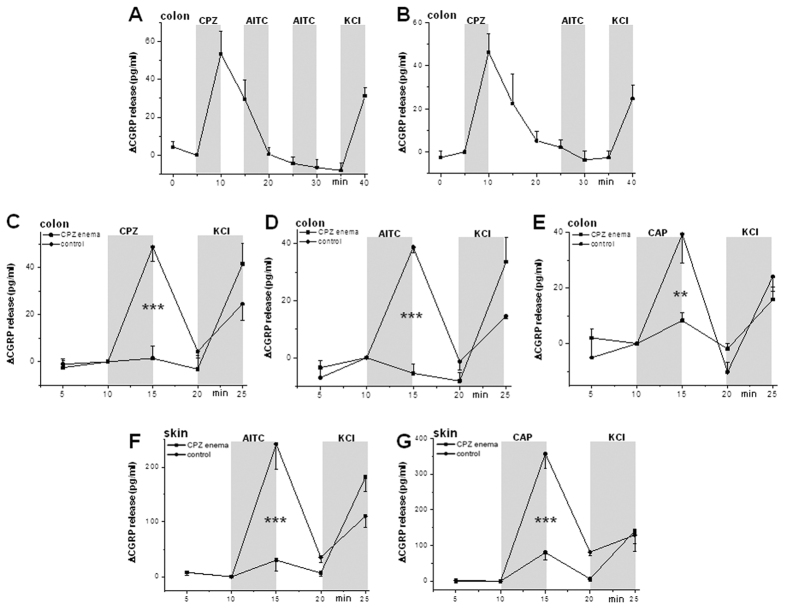
Local and systemic desensitization of peptidergic sensory nerves by capsazepine (CPZ) indicated by altered release of calcitonin gene-related peptide (CGRP). (**A**) Acute CGRP release from isolated colon preparations of WT mice induced by CPZ (100 μM); subsequent mustard oil (AITC, 100 μM) exposures fail to induce CGRP release, while unspecific depolarization by KCl (60 mM) is effective as normal. Data are means + SEMs (n = 8). (**B–D**) CPZ (100 μM)- and AITC (100 μM)-induced colonic CGRP release was abolished in mice pretreated with twice daily CPZ enemas for 7 d until the day before the release experiment, whereas CAP (1 μM)-induced colonic CGRP-release was strongly reduced but not abolished in these mice compared with controls. (**P < 0.01, ***P < 0.001, Mann Whitney U-test, each n = 6). (**E**) Similarly, AITC (100 μM)-induced CGRP release was abolished in isolated skin preparations from the hindpaws of mice pretreated with CPZ enemas. (**F**) In contrast, CAP (1 μM)-induced CGRP release was strongly reduced from the skin of these mice compared with controls. (**P < 0.01, ***P < 0.001, Mann Whitney U-test, each n = 6). Note that all KCl (60 mM) responses following desensitized CPZ and AITC responses were normal (**B**,**C**,**E**), whereas the KCl responses of the incompletely desensitized CAP-stimulated, neuron population (**D**,**F**) were as much reduced as in all control experiments, suggesting CGRP store depletion which is prevented by effective desensitization of the CPZ/AITC sensitive neuron subpopulation.

We then asked the question of whether the desensitization of whole animals by CPZ could be reproduced on the cellular level. To this end, we performed calcium-imaging experiments with isolated DRG neurons that had been obtained from control mice and from animals treated for 7 days twice daily with CPZ enemas. These neurons were necessarily kept in culture for 16 to 24 h, in the presence of NGF. A total of 399 neurons from control mice and 584 neurons from enema-treated mice were loaded with Fura-2 and calcium transients evoked by AITC, carvacrol, CAP and a KCl-rich solution were recorded. The responses to these TRPA1 (AITC and carvacrol) and TRPV1 (CAP) agonists were not significantly different in DRG neurons from control animals and enema-treated animals (Fig. S1). However, TRPA1 mRNA expression (qPCR) was about two fold upregulated in lumbosacral DRG prepared immediately after the final CPZ enema (Fig. S2) whereas no change in TRPA1 expression was detected when 24 h had elapsed *in vivo* after the final enema. TRPV1 mRNA did not change under both circumstances. Thus, transcriptional upregulation of the TRPA1 gene expression had taken place, due to the multiple CPZ enemas, but was rapidly reversed when the CPZ supply was discontinued. Under DRG culturing conditions, the same epigenetic reversal had likely taken place and all residual CPZ was probably washed-out, so that no residual desensitization could actually be expected. As the cellular models did not reflect the outlasting CPZ-induced desensitization of the whole animals *in vivo*, we looked for another strong indicator of the surprising systemic effect. The majority of nociceptive neurons is peptidergic, expressing predominantly calcitonin gene-related peptide (CGRP) and substance P (SP)^15^,^17^. Upon depolarization, as by KCl, and calcium influx, due to activation of voltage-gated calcium channels, these neuropeptides are released from the nerve fibers in a quasi-efferent function (neurogenic inflammation). On the other hand, activated TRP channels are very good calcium conductors by themselves and do not require support by any voltage-gated sodium or calcium channels in order to evoke vesicular exocytosis of CGRP^18^. Thus, stimulated release of CGRP can serve as an index of (peptidergic) nociceptor activation. By the same token vasoactive neuropeptides have various effects on the inflammation process per se, and depletion or desensitization of the peptidergic sensory neuron population has been shown to attenuate colitis^19^,^20^,^21^. To determine whether CPZ (531 μM) enemas desensitize through TRPA1, locally and systemically, we employed isolated mouse colon and skin preparations. Colons of healthy C57BL/6 mice (n = 8) were exposed to CPZ (100 μM) which induced massive CGRP release (Fig. 5); subsequent applications of AITC (100 μM) 5 min or 15 min after CPZ (Fig. 5A,B) could not anymore induce CGRP release, suggesting a profound acute functional cross-desensitization to the TRPA1 agonist. This effect cannot be due to depletion, as the ensuing KCl (60 mM) response was normal. Colons from mice that had been repeatedly treated with CPZ (531 μM) enemas *in vivo*, twice daily for 7 d were isolated and tested 12–16 h after the last enema. Neither CPZ (100 μM) nor AITC (100 μM) induced any CGRP release in this condition (Fig. 5C,D); notably, CAP (30 nM)-induced CGRP release was strongly reduced but not abolished (Fig. 5E). In contrast, KCl (60 mM)-induced CGRP release by unspecific depolarization was not only unreduced but actually enhanced after the CPZ enemas. This indicated that the colonic nerve fibers were not depleted of CGRP but rather overfilled, yet essentially desensitized in a sustained manner to chemical activation through TRPA1 as well as TRPV1 (n = 6). Finally, also the skin preparation, isolated 12–16 h after the last CPZ enema, showed that AITC (100 μM) and CAP (1 μM)-induced CGRP release was strongly reduced in accordance with the body-wide desensitization observed in the behavioral tests (Fig. 5F,G, see Fig. 4A–F) (n = 6).

## Discussion

It has been assumed that inhibition of TRPV1 in peptidergic colonic sensory neurons by CPZ attenuates colitis through reducing neuropeptide release and neurogenic inflammation. In stark contrast, we found that the activation of TRPA1 by CPZ followed by profound and sustained desensitization caused the anti-inflammatory effect. More importantly, we discovered an unexpected powerful impact of CPZ via TRPA1 on peripheral nociception in general. To the best of our knowledge the present study is the first to demonstrate body-wide nociceptor desensitization through TRPA1.

### TRPA1 and neuropeptide release in inflammation

Previously, one dermatological study suggested that local TRPA1 desensitization by AITC suppressed the inflammatory reaction in an allergic contact sensitivity model of the mouse^22^. If the skin was topically pretreated with AITC (1%) or capsaicin (0.5%) or injected with a CGRP receptor antagonist mice sensitized to electrophilic fluorescein isothiocyanate (FITC) showed a reduced ear swelling response upon repeated FITC challenge. In a murine model of allergic asthma, TRPA1 in sensory neurons mediated leukocyte infiltration and increased pulmonary mucus production and airway hyperreactivity, supposedly through the release of neuropeptides such as CGRP and SP^12^. Only recently, TRPA1 was ascribed a crucial role in the context of gram-negative bacterial infections. TRPA1 in nociceptive sensory neurons not only mediated LPS-induced pain but also vascular reactions including neurogenic inflammation (CGRP release)^16^. We recently supported the role of TRPA1 as a gatekeeper for inflammation when we demonstrated that activation and sustained sensitization of TRPA1 by various endogenous mediators of inflammation leads to increased release of the sensory neuropeptides. This initiates and maintains trinitrobenzene sulfonic acid (TNBS) and DSS colitis in mice, respectively^6^,^14^. Also a third model of mouse colitis, induced by an oxazolone enema after presensitization, was found to depend on neuropeptide release^23^. Previously, two studies reported therapeutic effects of repeated CPZ enemas on DSS and TNBS colitis in rats through putative inhibition of TRPV1 and, thus, reduced neuropeptide release from peptidergic colonic sensory neurons^7^,^8^. Repeated intraperitoneal injections of CPZ in DSS colitis and oral administration of another TRPV1 antagonist (JNJ 1085734) also appeared therapeutically effective^9^. However, when studys employed TRPV1-deficient mice they reported results that were contradictory. TRPV1-deficient mice receiving 5% DSS via drinking water developed severe colitis to nearly the same degree as WT mice^10^. Even increased colitis susceptibility was reported from TRPV1-deficient mice challenged with dinitrobenzene sulfonic acid (DNBS)^11^. The findings of the present study suggest that the molecular mechanism behind (long-term) colitis protection by CPZ is the sustained (over)stimulation and consecutive desensitization of TRPA1 and TRPA1/TRPV1 co-expressing neurons rather than antagonism to TRPV1, since TRPV1-deficient mice were as well-protected by CPZ enemas as were WT mice. CPZ enemas led to a profound desensitization of TRPA1-expressing neurons which reduced neuropeptide release and neurogenic inflammation.

### Molecular mechanisms of CPZ action

Our patch clamp and calcium imaging measurements demonstrate a concentration-dependent activation of heterologously expressed human TRPA1 by CPZ. Activation of TRPA1 by electrophiles, such as AITC or TNBS, occurs through covalent modification of cysteine residues in the N-terminal domain of the channel^4^,^6^. By using the triple cysteine mutant hTRPA1-3C (C621S, C641S, C665S), we were able to identify high-affinity CPZ binding to the same site as AITC, which is consistent with the electrophilic nature of CPZ. However, the 3C motif was no longer required for TRPA1 activation at 100 times higher CPZ concentration. The fact that large, saturating concentrations of CPZ are able to activate the hTRPA1-3C mutant (in which cysteines at positions 621, 641 and 665 have been replaced with serines) is not surprising, as it has been shown that this mutant channel is not completely insensitive to electrophilic compounds, but merely less sensitive, leading to a pronounced rightward shift in the concentration dependence of activation by methylglyoxal^24^. Finally, our experiments with saturating concentrations of N-acetyl cysteine (NAC) demonstrated that CPZ acts as an electrophilic agonist for hTRPA1.

The calcium microfluorimetry measurements in lumbosacral DRGs showed an almost complete overlap (93%) of the chemosensitivity to CPZ and to AITC at comparable concentrations. As in several recent publications the overlap between capsaicin- and AITC-sensitive DRG neurons was not as complete as erstwhile postulated^25^,^26^,^27^, however, 78% of the neurons responding to both prototypical agonists were also activated by capsazepine. This high percentage may account for the efficiency of chronic CPZ to induce general nociceptor desensitization. Of note, all previously reported off-target effects of CPZ were related to blocking other ion channels than TRPV1 at higher (>50 μM) concentrations: nicotinic acetylcholine receptors, voltage-gated calcium channels, transient receptor potential melastatin 8 (TRPM8), and the hyperpolarization-activated HCN^1^,^2^,^3^,^4^ channels^28^,^29^,^30^,^31^. Only the block of TRPM8 by CPZ was reported to mediate an anti-nociceptive effect in a behavioral model of neuropathic hypersensitivity to cold stimulation^32^. The massive off-target effect of CPZ on TRPA1 (at 0.1–50 μM) that we demonstrate is contrary to the original pharmacological claim to “antagonize sensory neuron excitation” (by CAP)^33^. In fact, CPZ excited sensory neurons sensitive to CAP (see above), and it did so by activating the universal chemoreceptor channel TRPA1 that is co-expressed in a large subpopulation of TRPV1-positive nociceptive neurons, which are specialized for sensing potentially damaging, noxious stimuli of any modality that can evoke pain in humans. In addition, the transient activation of TRPA1 by CPZ was followed by a particularly profound and sustained desensitization of the neurons to TRPA1-specific and to TRPV1 stimuli, such as heat (cross-desensitization). Such desensitization also resulted from the treatment of mice *in vivo* with repeated CPZ enemas and CPZ or AITC in the drinking water.

### Capsazepine-induced TRPA1-mediated effects are specific

The sustained desensitization of stimulated CGRP release from isolated skin and colon after CPZ enemas cannot be attributed to a disruption of the vesicular exocytotic release mechanism, because this remained undisturbed as evidenced by KCL-induced unspecific depolarization. Thus, the excitability with respect to the function of voltage-gated calcium channels in the sensory nerves was well maintained. Excitability with respect to action potential triggering does not matter in our models, as TRP channel-induced CGRP release cannot be altered even by TTX and lidocain^18^,^34^. However, in additional experiments we could not recapitulate the sustained desensitization in the cellular model of cultured DRG neurons, employing calcium imaging to compare CPZ enema-treated and control donors. Both the numbers of AITC responsive neurons and the magnitudes of the AITC, carvacrol, capsaicin and KCl-evoked calcium transients were about the same in both groups. We assume that the invasive and time consuming culturing procedures cleared all residual CPZ and/or extinguished its desensitizing effect. *In vivo*, the lipophilic drug may be better retained in the tissues or re-distributed. Some support for this interpretation comes from additional experiments quantifying the mRNA for TRPA1 and TRPV1 in DRGs. The ganglia were either isolated and processed immediately after the final CPZ enema or 24 h later. TRPA1 gene expression was clearly enhanced in the former case but normal after one day of survival, indicating a rapid reversal of the epigenetic effect. Our behavioral experiments still indicate that CPZ induced a TRPA1-driven local desensitization of colonic sensory neurons, since TRPA1-deficient mice did not show the initial nocifensive responses during and after the CPZ enema administrations. By contrast, WT mice gradually lost their pain responses (writhing, VMRs). Intriguingly, desensitization did not remain a local, colonic phenomenon but was extended – supposedly by absorption and systemic distribution of the drug – to such distant organs as the eye, where drops of CAP or AITC solution were no longer able to evoke the eye-wiping reaction, or as the skin, where these irritants induced only minimal neuropeptide release. The results from the radiant heat plantar test after oral administration of CPZ and AITC further support the notion of a body-wide, general desensitization of nociceptive nerve endings via TRPA1 without affecting mechanosensitivity to non-noxious stimuli. Since AITC application via drinking water had effects similar to those after oral administration of CPZ, we suggest a class effect of TRPA1 agonists. It remains possible that the stronger effect of CPZ compared with AITC was due to the simultaneous TRPV1 antagonism of CPZ.

### Capsazepine-induced neuronal desensitization - a well-tolerated therapeutic approach

As a secondary finding, the feasibility of the oral administration route of CPZ and AITC, its apparent tolerability, safety, and efficacy can be postulated. Higher concentrations of CPZ may even support the anti-nociceptive effect of the chronic desensitization. Even the original target effect of CPZ, blocking the CAP binding site in TRPV1, may be adjuvant because it would preclude effects of inflammatory endovanilloids^35^. A general desensitization of nociceptive nerve endings could also be achieved by systemic administration of CAP or resiniferatoxin (repeated over days with incremental doses)^6^,^36^. However, this TRPV1-mediated desensitization is as profoundly effective as certain TRPV1-blocking drugs tested in humans, which lead to a loss of noxious heat sensitivity associated with the hazard of burn injury or scalding, or other TRPV1 antagonists that induce hyperthermia^37^,^38^. In addition, TRPV1-mediated desensitization inevitably results in a complete depletion of neuropeptides, such as CGRP and SP, which also are essential trophic factors for tissue integrity and repair^36^. In contrast, CPZ-induced chronic desensitization through TRPA1 fully preserved neuropeptide storage and the ability of the nerve to release CGRP upon depolarization; it just prevented chemically–induced neuropeptide release.

## Conclusions

Our findings provide novel insight into the molecular mechanisms that lead to colitis attenuation by CPZ and strengthen the crucial role of TRPA1 as a gatekeeper for inflammation. Moreover, we demonstrate a novel mechanism to induce body-wide anti-nociception through TRPA1 desensitization. This dual mechanism may provide new therapeutic options not only for patients with inflammatory or irritable bowel disorders but also for patients suffering from other chronic states of pain or inflammation. TRPA1 desensitization could not only provide symptomatic relief but it could also support real cure, potentially enabling CPZ or derived novel “TRPA1 desensitizers” to become disease-modifying-drugs. Subjects with demonstrated genetic susceptibility to inflammatory or irritable bowel disorders could even benefit from a preventive approach.

## Materials and Methods

For details see *SI* Materials and Methods. All animal experiments were performed in accordance with the relevant guidelines and regulations approved by the Animal Protection Authority, District Government Mittelfranken, Ansbach, Germany.

### Patch clamp experiments

Whole-cell voltage clamp experiments were carried out in HEK293 cells transiently transfected with human TRPA1 (hTRPA1) using an Axon Axopatch 200B patch clamp amplifier and the pCLAMP 10 software (Molecular Devices, Sunnyvale, CA, USA).

### Ratiometric Ca^2+^-imaging measurements

Mouse lumbosacral DRG neurons were isolated and cultured as previously described, using nerve growth factor (100 ng/mL mouse NGF 2.5 S; Alomone Labs, Jerusalem, Israel)^6^.

### Induction of experimental colitis and monitoring

Experimental colitis was induced by Dextran Sulphate Sodium (DSS, 5%, molecular weight: 36.000–50.000, MP Biomedicals, Illkirch, France) which was added to the drinking water for 7 days. Monitoring of the course of colitis was performed by daily body weight measurements and mouse colonoscopies on day 2, 4 and 6. To assess colitis severity, previously established endoscopical and histological score systems were used^6^,^39^.

### Capsazepine enemas and drinking water supplementation

Mice received capsazepine (CPZ, 300 μl, 531 μM) or vehicle (PBS) enemas twice daily for seven days under brief isoflurane anesthesia. In another experiment the same concentration of CPZ (in 0.1% DMSO) or AITC (500 μM) (in 0.1% DMSO and 0.1% Kolliphor, Sigma-Aldrich) was continuously applied through the drinking water.

### Behavioral experiments

All experiments were done in compliance with the local Animal Protection Authority (Approval No. 54–2532.1–33/12, Government of Mittelfranken, Ansbach, Germany). Animals were randomized and investigators were blind to the different groups of animals and the handling of data (treatment vs. control). The writhing test, eye-wipe, von-Frey and Hargreaves’ test as well as measurements of visceromotor responses were performed as published before^40^,^41^,^42^,^43^.

## Additional Information

**How to cite this article**: Kistner, K. *et al*. Systemic desensitization through TRPA1 channels by capsazepine and mustard oil - a novel strategy against inflammation and pain. *Sci. Rep.* **6**, 28621; doi: 10.1038/srep28621 (2016).

## Supplementary Material

Supplementary Information
