# Supplementary material for: Systemic desensitization through TRPA1 channels by capsazepine and mustard oil - a novel strategy against inflammation and pain
Source: Sci Rep. 2016 Jun 30;6:28621. doi: 10.1038/srep28621 (PMC4928060; doi:10.1038/srep28621)
Supplement: Supplementary Information [file srep28621-s1.doc]

**SUPPLEMENTARY INFORMATION**

**Systemic desensitization through TRPA1 channels by capsazepine and mustard oil - a novel strategy against inflammation and pain**

**Short title: TRPA1 desensitization attenuates colitis and pain**

Katrin Kistner^1^, Norbert Siklosi^1^, Alexandru Babes^2^, Mohammad Khalil^1,3^, Tudor Selescu^2^, Katharina Zimmermann^4^, Stefan Wirtz^3^, Christoph Becker^3^, Markus F. Neurath^3^, Peter W. Reeh^1^, Matthias A. Engel^3*^

KK, NS and AB contributed equally

^1^Institute of Physiology and Pathophysiology, Friedrich-Alexander-Universität Erlangen-Nürnberg, Universitätsstr. 17, 91054 Erlangen, Germany. ^2^University of Bucharest Department of Physiology, Faculty of Biology, Splaiul Independentei 91-95, 050095 Bucharest, Romania. ^3^Department of Medicine 1, Universitätsklinikum Erlangen, Ulmenweg 18, 91054 Erlangen, Germany. ^4^Department of Anaesthesiology, Universitätsklinikum Erlangen, Krankenhausstr. 12, 91054 Erlangen, Germany.

**Supplementary Materials and Methods**

**Chemicals and solutions**

Capsazepine, capsaicin, allyl isothiocyanate, carvacrol and ionomycin (all purchased from Sigma-Aldrich, Taufkirchen, Germany) were dissolved in pure ethanol obtaining 0.1-1 M stock solutions. All drug solutions were prepared immediately before each experiment from deep-frozen aliquots of stock solutions by dilution with synthetic interstitial fluid (SIF) (CGRP-release measurements) or external solution (calcium-imaging, patch-clamping) to the final concentration. SIF comprised (in mM) 107.8 NaCl, 26.2 NaCO3, 9.64 Na-gluconate, 7.6 sucrose, 5.05 glucose, 3.48 KCl, 1.67 NaH2PO4, 1.53 CaCl2 and 0.69 MgSO4, gassed with 95% oxygen and 5% carbon dioxide to pH 7.4. The standard external solution used for both patch clamp and calcium microfluorimetry contained (in mM): NaCl 145, KCl 5, CaCl_2_ 1.25, MgCl_2_ 1, HEPES 10, glucose 10 (pH 7.4 with NaOH; osmolarity, 308-312 mOsm). The pipette solution contained (in mM): K-gluconate 135, NaCl 4, MgCl_2_ 3, Na_2_ATP 5, Na_3_GTP 0.3, EGTA 5, HEPES 5 (pH 7.25 with KOH; osmolarity 280 mOsm).

**Statistical analysis**

Results are presented as mean ± standard error of the mean (SEM). The number (n) that is quoted throughout the manuscript refers to the number of animals used per experiment. Applied statistical tests and levels of significance are denoted in the figure legends.

**Animals**

Male WT C57BL/6 mice and global null mutant mice for TRPV1 and TRPA1 were used in these experiments (6-10 weeks). TRPV1 and TRPA1 mutants have been continuously backcrossed to C57BL/6 mice for more than ten generations and were fully congenic. Initial breeding pairs of TRPV1^+/–^ and TRPA1^+/–^ mice were donated by Dr. Davis (formerly Glaxo Smith Kline, Harlow, UK) and Drs Kwan and Corey (Harvard, Boston, MA), respectively. Commercially available primers (Metabion, Martinsried, Germany) were used for genotyping. WT and knockout alleles were identified with two genomic 5’ primers. The sequences were CATGGCCAGTGAGAACACCATGG and AGCCTTTTGTTCTTGGCTTCTCCT for the Trpv1 WT allele and CCGGTGCCCTGAATG-AACT and AAGACCGGCTTCCATCCGA for the knockout allele; CCTCGAATCGTGGATCCA-CTAGTTCTAGAT and GAGCATTACTTACTAGCATCCTGCCGTGCC for the Trpa1 knockout allele and TCCTGCAAGGGTGATTGCGTTGTCTA and TCATCTGGGCAACAATGTCACCTGCT for the respective wildtype allele. Keeping and handling of the animals was in accordance with the German Animal Protection Laws controlled and approved by the veterinary authorities of the district government, Regierung von Mittelfranken, Ansbach, Germany (Approval No. 54-2532.1-33/12).

**Quantitative PCR of TRPA1 and TRPV1 expression**

TRPA1 and TRPV1 mRNA expression was measured in murine DRG neurons either isolated and processed immediately after the final CPZ enema (after one week of twice daily enema administration) or 24 h later. RNA was reverse transcribed by using iScript™ cDNA Synthesis kit (Bio-Rad, München, Germany), as controls random samples without reverse transcriptase (No-RT) were used. Quantitative PCR was performed in duplicate by using the Sensimix SYBR No-Rox kit (Bioline, Luckenwalde, Germany) in a Mastercycler, realplex (Eppendorf, Hamburg, Germany). Mouse qPCR HPRT, TRPA1, TRPV1 primers were ordered from (Qiagen, Hilden, Germany; Cat. no.: QT00166768, QT00133791, QT00167048). The relative quantification of the genes of interest was determined in relation to the housekeeping gene hypoxanthine-guanine-phosphoribosyl-transferase (HPRT). Finally, the ΔΔCq method was used to investigate the differences in the gene expression levels.

**Heterologous expression**

HEK293t cells were transfected as previously described with human TRPA1 or rat TRPV1 cDNA (6). After incubation for 12-15 h, cells were plated on poly-D-lysin (200 µg/ml; Sigma-Aldrich) coated sterile glass cover slips and used for experiments within 24 hours. Only one cell was used for recording from each dish.

**Patch clamp experiments**

The current was lowpassed at 1 kHz and sampled at 2 kHz. Patch pipettes were pulled from borosilicate glass capillaries (TW150F-3, World Precision Instruments, Berlin, Germany) at a final resistance of 1.7 – 5.0 MΩ. The holding potential was -60 mV and in some experiments voltage ramps from 100 to +100 mV of 400 ms duration were applied every 4 s.

**Ratiometric Ca^2+^-imaging measurements**

Ratiometric [Ca^2+^]_i_ measurements on cultured DRG neurons and transfected HEK293t cells were done using the fluorophor Fura-2 AM (5 µmol/L). Coverslips with attached cells were incubated for 30 min at 37 °C in standard extracellular solution (see above) containing 5 µM Fura-2 AM and 0.02% Pluronic (both from Invitrogen, Carlsbad, CA, YSA) and then left to recover for 15 min before recording. Following this procedure, coverslips were mounted on an Olympus IX71 inverted microscope stage and imaged with a 10 x objective and an Olympus IX 73 inverted microscope equipped with a TILL Photonics Polychrome V monochromator, respectively. Cells were permanently superfused with external solution using a software-controlled gravity-driven 6-channel common-outlet perfusion system. The fluorescent calcium indicator was excited at 340 and 380 nm (5 ms exposure time) with a Polychrome 5 monochromator (Till Photonics, Gräfelfing, Germany) and images were acquired at 1 kHz with a 12 bit CCD camera (Imago Sensicam QE, Till Photonics, Gräfelfing, Germany) and the TILLvisION 4.0.1.3 software (also used for off-line analysis). Data were recorded as the ratio between the fluorescence emitted when the dye was excited at 340 nm, and at 380 nm, respectively (F340/F380). Background fluorescence intensity was subtracted before calculating the ratios. A cell was considered responsive to a chemical agonist if the average fluorescence ratio (F340/F380) measured for the interval between the application of the agonist and the subsequent chemical stimulus was larger than the average fluorescence ratio measured for the 10 data points immediately before stimulation by more than 10%.

**Histological evaluations**

Colonic segments were rinsed with saline and placed in 10% neutral buffered formalin. Segments were then embedded in paraffin, sectioned (5 µm) and stained with hematoxylin/eosin. Samples were examined by light microscopy (Olympus BX-50, Melville, NY, USA). Scoring was performed by an investigator, blinded to the experimental groups. Photomicrographs were taken with a Zeiss LSM 780 microscope using a 20x objective and an AxioCam digital camera (Carl Zeiss Microimaging GmbH, Göttingen, Germany).

**Capsazepine enemas and drinking water supplementation**

The concentration of the capsazepine enemas (531 µM) was chosen based on previous publications that showed a therapeutic effect. Two previous publications used CPZ enemas with a concentration which was supposedly ≥ 400 µM, however, the concentration of the CPZ enema was only exactly calculable in the report of Kihara et al. (2003) (531 µM) (8), thus, is the one that is used in the present study. Fujino et al. (2004) used CPZ enemas at a dosage of 1 µmol/kg body weight (BW)/ml (7). Another publication used intraperitoneal injections at a dosage of 2.5 mg/kg BW, thus, the CPZ concentration was supposedly about 400 µM (9). During the whole colonic or peroral CPZ treatment periods mice did not show any obvious pathology or altered behavior, indicating tolerability and a possible lack of toxicity of chronic CPZ. They did not show any signs of dehydratation, such as exsiccation, reduced locomotor activity or body weight loss. The same good tolerability accounts for AITC (500 µM) supplementation via drinking water. Peter W. Reeh and Matthias A. Engel tested both drinking water solutions by themselves: CPZ (531 µM) was found to only have a slightly bitter taste. AITC at 500 µM evoked a mild burning sensation especially in the retrolabial oral mucosa. To our knowledge, there is no data about bioavailability of these two compounds, however, to justify the use of similar concentrations, *in vitro* they show about the same EC_50_ values with regard to TRPA1 agonism. The substantial drug effects of both compounds shown in the Results section suggest a sufficient bioavailability on both routes of administration.

**Behavioral experiments**

*Writhing test*

Immediately after application of each capsazepine enema mice were observed and the number of writhing of each mouse was counted within a period of 20 min. Contractions of the abdominal muscles or characteristic stretching of the hind limbs were counted as writhing behavior as previously published (40).

*Eye-wipe test*

The test was performed 12-16 hours after the final CPZ enema. One drop (20 µl) of a capsaicin (CAP, 1 mM) or mustard oil (AITC, 100 µM) solution was applied into the right or left eye of the mouse. The contralateral eye served as a vehicle (PBS) control. Immediately after application mice were observed for 60 s and eye-wipes were counted. Eye-wipe behaviour differed typically from grooming as published before (41).

*Visceromotor responses*

Three days before VMR measurements electrodes were implanted in mice under isoflurane anesthesia and metamizol(dipyrone) (200 mg/kg BW i.v.). During the 48 hours post-surgery mice received i.p. metamizol injections (200 mg/kg BW) three times a day. Electrodes were made by stripping 3 mm of insulation from the ends of teflon-coated stainless steel wires (Science Products GmbH, Hofheim, Germany). Tunneling of electrodes to the dorsum of the neck was necessary to avoid cropping. During the recovery period of three days, mice were acclimatized to the recording chamber. Visceromotor responses (VMR) were quantified by measuring the integrated electromyographic (iEMG) recordings of the abdominal musculature. EMG activity was normalized by integrating EMG signals (ΔiEMG in µV x s) recorded during the 10 s before enema application and compared to those signals that were recorded within 20 s after the start of enema application; enema application itself took about 10 s. On the day of testing, mice were lightly anesthetized by isoflurane inhalation and restrained in a custom-made clear polycarbonate tube. Enemas were applied through a small plastic catheter that was smoothly inserted transanally so that the tip of the catheter was placed 5 cm from the anus, secured by tape to the tail. EMG activity of the external oblique muscles was amplified and filtered using the Bridge Amp ML221 amplifier; data were recorded and analyzed on- and offline using LabChart (both ADInstruments, Colorado Springs, USA). At the end of each experiment mice were killed in a 100% carbon dioxide atmosphere by cervical dislocation.

*Hargreaves’ and von Frey test*

Paw withdrawal latencies and thresholds were measured in response to radiant heat stimulation and mechanical stimulation, respectively (43). Animals were habituated to the testing environment for each 60 min on three separate days. Before the beginning of each test day mice were acclimatized in the testing environment for 30 min. Mice were subject to repeated plantar radiant heat or mechanical stimulation through the meshes of a grid floor on which the mice were allowed to move unrestrictedly. Mechanical thresholds were determined using a Dynamic Plantar Aesthesiometer (Ugo Basile, Varese, Italy). The von Frey–type filament of 0.5 mm diameter exerted increasing force (g) to the plantar surface of the hindpaw until the animal twitched its paw. The Hargreaves’ test was performed employing a beam of radiant heat of relatively high, constant, intensity (7 of 9 arbitrary units) which was focused to the plantar side of the hindpaw. To determine thermal thresholds, the beam was automatically switched off upon withdrawal of the paw. An interval of 60 min was allowed between the two sets of tests. Both left and right paws were measured repeatedly with at least 3 min between stimuli and paw withdrawal latencies from both paws were analyzed separately.

**Stimulated CGRP release from the isolated mouse colon and skin**

CPZ-treated animals were used 12-16 hours after the final enema. Mouse colon and hairy hindpaw skin were isolated immediately after killing the animals in a rising (to 100%) carbon dioxide atmosphere by cervical dislocation. The excised mouse colon was flushed clean with PBS and tied at both ends. Colon and skin were fixed with surgical silk on small acrylic rods to assure minimal mechanical manipulation during organ transfer from one tube to the other. For stimulation with a high concentration of potassium, which served as an unspecific depolarizing stimulus, a modified SIF solution was used containing 60 mM KCl (0.447g KCl/100ml) and a reduced concentration of NaCl (0.3g/100ml) to maintain osmolarity. Briefly, a series of four glass tubes were each filled with 800 µl of SIF and placed in a temperature-controlled shaking bath (37.5 °C). Isolated mouse colon or skin were incubated in each tube for 5 minutes; periods with chemical stimulation are indicated in the figures. iCGRP content was measured from 100 µl samples of the diluents using a commercial CGRP kit (Bertin Pharma, Montigny le Bretonneux, France). EIA plates were measured photometrically using a microplate reader (Dynex, Germany).

**Supplementary Figure Legends**

**Supplementary to Fig. 3.**

Almost all CPZ-sensitive neurons were also activated by the selective TRPA1 agonist AITC (185/200; 93%). Of 301 AITC-sensitive neurons 185 were activated by CPZ (61%). In comparison, of 605 AITC-insensitive DRG neurons only 15 were activated by CPZ (2.4%). A substantial fraction of AITC-sensitive neurons (125/301, 41%) were not capsaicin-sensitive. While this appears to be a surprisingly large fraction, expression of TRPA1 in TRPV1-negative DRG neurons has previously been reported (40). Moreover, before being challenged with capsaicin these cells had been exposed to relatively large concentrations of capsazepine (TRPA1 agonist and TRPV1 antagonist) and AITC (TRPA1 agonist), and therefore a certain amount of cross-desensitization and/or residual inhibition may be expected, thus reducing the apparent fraction of capsaicin-sensitive neurons.

**Supplementary Fig. S1.**

DRG neurons from control (black traces, n = 399 cells; n = 3 mice) and enema-treated (red traces, n = 584 cells; n = 3 mice) mice were stimulated with allyl isothiocyanate (AITC, 100 µM, 30s), carvacrol (100 µM, 30s), CAP (1 µM, 20s) and high KCl (60 mM, 30s). The interval between stimuli was 4 min, with the exception of the interval between CAP and KCl applications, which were 3 min. Calcium ion influx is illustrated as mean (straight lines) ± SEMs (dotted lines). 193/399 (48%) DRG neurons from control mice were AITC-sensitive and 284/585 (49%) neurons from CPZ-treated mice were AITC-sensitive.

**Supplementary Fig. S2**

Relative expression of TRPA1 and TRPV1 mRNA in lumbosacral dorsal root ganglia taken from mice either 5 min or 24 h after the final CPZ enema compared with vehicle-treated controls (**P* < 0.05, Mann Whitney U-test, each n = 4).
